# Case Report: Feminizing the Male Face

**Published:** 2009-01-09

**Authors:** Mohammad Ghasem Shams, Mohammad Hosein Kalantar Motamedi

**Affiliations:** ^a^ Assistant Professor, Department of OMF Surgery, Baqiyatallah Medical Center; ^b^ Professor of OMF Surgery, Trauma Research Center, Baqiyatallah Medical Sciences University, Tehran, Islamic Republic of Iran

## Abstract

**Objective:** Sex reassignment surgery is accepted in properly planned cases to change facial appearance in accordance with a new gender. Although changing the hairdo or makeup, and depilation, give satisfying cosmetic results in many patients, hard and soft tissue facial surgeries are needed in most cases. Unfortunately, few studies on facial corrections have been published and feminizing a male face is still considered an arbitrary undertaking. We present a useful surgical protocol to feminize the male face. **Method:** Ten male-to-female transsexuals aged 20 to 32 years (average 23 years) were referred after gender reassigning for facial feminization (1990–2007). Clinical examination of the patients revealed variable degrees of common masculine features such as long square faces, broad chins, excessive projection, frontal bossing, prominent flaring gonial angles, high hairlines, and low-set eyebrows. After complete clinical assessment and paraclinical workup including cephalometry, the patients were admitted for feminizing surgery. The face was outlined in geometric proportions. It was aimed to round the male face using a sequence of staged surgical procedures for both hard and soft tissues. **Results:** Ten male patients aged 20 to 32 years (average 23 years) underwent facial feminization using basic surgical guidelines. Various amounts of soft and hard tissue changes were required for individual patients and were tailored for each case according to the preoperative workup and standard parameters. **Conclusion:** All 10 patients were satisfied with their improvement in appearance; the degree of impact these procedures may have on lifestyle remains to be assessed.

The sex of a person is what one sees while gender is what one feels.^1^ Classic tanssexualism is a gender identity disorder in which there is strong and ongoing cross-gender identification and a desire to live and be accepted as a member of the opposite sex.^1^–^8^ However, only about 10% to 15% of patients with gender dysphoria fall into this category.^1^ In these patients, there is persistent discomfort with the anatomic sex and a lack of appropriateness in the gender role of that sex. There is also a wish to undergo somatic treatment to make the body as harmonious as possible with one's self-experienced gender identity. Although genital reassignment is the private domain of a transsexual, other, more visible body features such as the face can impede successful social acceptance as a member of the opposite sex.^1^ Despite the fact that facial features in transsexuals may seem to be of lesser importance than the reassignment of the genitalia, these are of utmost importance for passing as a member of the opposite sex in public. Facial surgery can help feminize both the facial bones and the soft tissues in male-to-female transsexuals,^2^,^5^–^10^ although exactly what surgical features feminize the face has been arbitrary. This article presents a staged surgical protocol for both hard and soft tissues to feminize the male face and parameters to consider prior to surgery.

## METHODS

**Figure 1:**
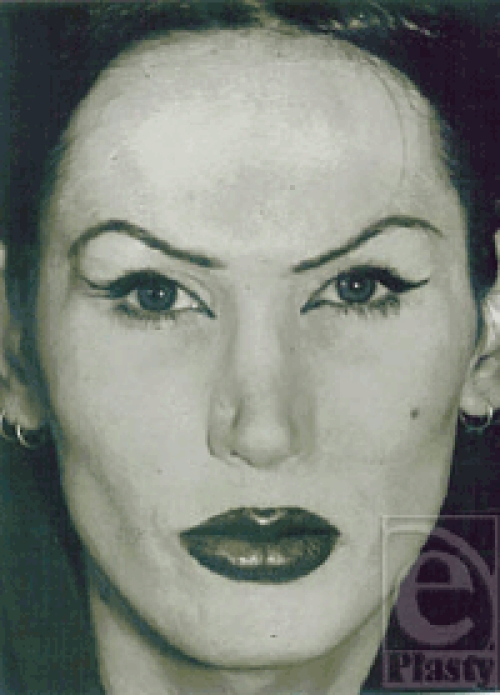
Facial view of a 23-year-old male-to-female transsexual before gender-reassigning facial correction.

**Figure 2:**
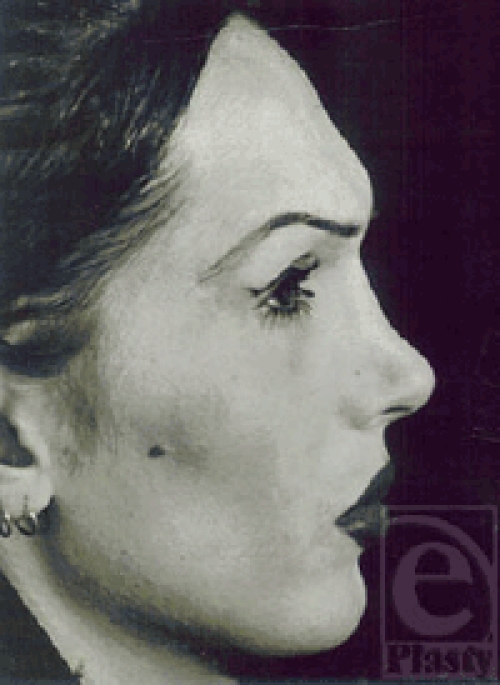
Lateral view of the patient. Note the long face, square chin, flared mandibular angles, and masculine features.

**Figure 3:**
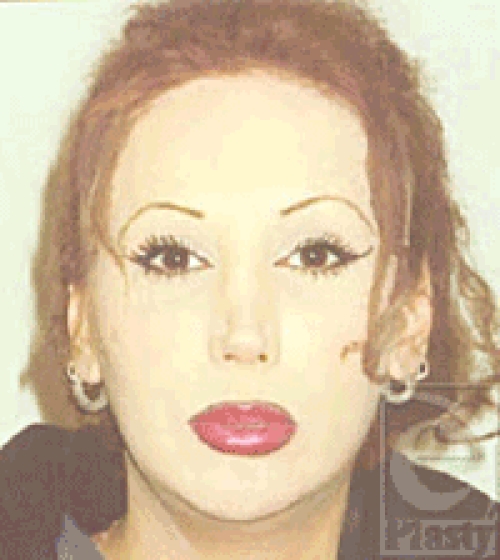
Facial view 4 years after facial correction via genioplasty setback and vertical reduction, gonial angles shaving, lateral and inferior cortical shaving of the entire mandible, zygoma advancement osteotomy, removal of buccal fat, revision rhinoplasty, supraorbital rim contouring and subcutaneous fat removal, frontal bossing shaving, eyebrow lifting, hairline lowering, and facial hair removal.

**Figure 4:**
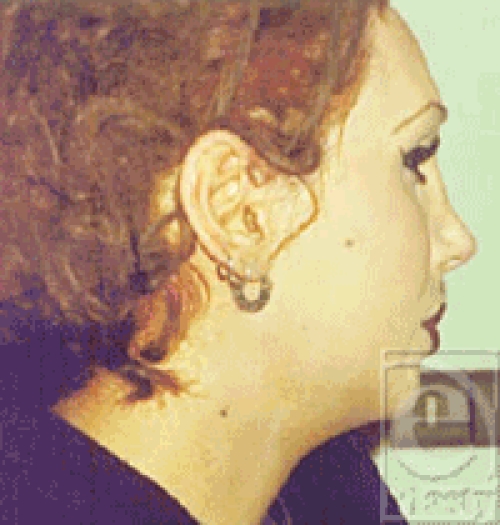
Lateral view of the patient.

Ten male-to-female transsexuals aged 20 to 32 years (average 23 years) were referred for gender-reassigning facial surgery and feminization from 1990 to 2007. Clinical examination in these patients revealed variable degrees of common masculine features such as long square faces and broad chins with excessive projection, frontal bossing, prominent flaring gonial angles with high hairlines and low-set eyebrows (Figs 1 and 2). After complete clinical assessment of the patients and paraclinical workup including cephalometric radiography and tracing, the patients were admitted for feminizing surgery. The standard procedures we used for feminization were as follows: to feminize the jaw, first under general anesthesia via a nasoendotracheal intubation, an intraoral degloving vestibular incision was made from left to right retromolar pads. After reflection of the mucoperiosteum and tunneling under the mental nerves, genioplasty setback and vertical reduction were performed. Then, the flared gonial angles were shaved laterally and rounded inferiorly using rotary instruments. Next, lateral and inferior cortical shaving of the entire mandible was performed to conform to the chin. Bilateral osteotomies of the zygoma and advancement were also done. Several months later, rhinoplasty was performed. In addition, through an incision in the hairline, undermining of the scalp, a 1.5- to 2.5-cm strip of skin was excised. Afterwards, supraorbital rim contouring with subcutaneous fat removal of the forehead, frontal bossing shaving, eyebrow lifting, and hairline lowering was performed. Later, facial hair removal was done using a laser (Figs 3 and 4).

## RESULTS

Our experience in treating 10 male-to-female transsexuals aged 20 to 32 years (average 23 years) correlated a set of effective, general, and surgical soft and hard tissue procedures and guidelines for facial feminization. Considerable feminization was achieved after these procedures in all our subjects. Various amounts of soft and hard tissue changes were required for individual patients depending on facial features and findings. Thus, all these procedures may not be required and also should be tailored for each individual case according to the preoperative workup and assessment and comparison with standard parameters (Table 1).

## DISCUSSION

Surgical corrections in male-to-female transsexuals have been somewhat arbitrary. Reports on this important topic in the literature are sparse. Clinical and radiographic findings must be correlated with the standard list of parameters as the basic guidelines to seek. It should be of note that these parameters may vary in various ethnicities and different societies.^2^,^5^–^10^ Surgical corrections of the facial hard and soft tissues that are used to change the facial appearance in male-to-female transsexuals more or less included mandibular angle and body reduction, genioplasty, bimaxillary osteotomies, increasing the zygomatic prominence, blepharoplasty, rhinoplasty, eyebrow lifting, hairline lowering, reducing frontal bossing, and laser hair removal.

The standard procedures we use for feminization are categorized and staged as follows:

*Reduction genioplasty*. Male patients with a broad or square chin with excessive projection will benefit from contouring the chin or genioplasty setback to increase convexity of the face. Because a small and rounded chin is regarded as feminine, thus, chin reduction in both a vertical and a horizontal direction is desirable.^1^,^3^

*Total mandibular angle and body reduction*. Because of a larger bony volume and thicker soft tissue covering, especially in the masseter muscle region, the lower border of the mandible and the gonial angle are more pronounced in men than in women. A pronounced mandibular angle is regarded a masculine feature.^1^–^3^ In the frontal view, pronounced mandibular angles can contribute to the square face associated with a masculine appearance. Reduction of these angles is, therefore, believed to be beneficial in feminizing a masculine face.^1^–^3^ Reduction of the total mandibular body, inferior border, and lateral aspect will yield favorable results.

*Maxillomandibular osteotomies*. Using the Le Fort I technique combined with a bilateral sagittal split osteotomy, the maxilla can be placed forward in combination with a dorsal impaction. This can make the face round and more convex and make the face look younger.^1^,^3^

*Advancement of the zygoma*. Advancement of the zygomatic complex creates more projection of the facial contours and is considered feminine. In addition to alloplastic onlays on the zygomatic bones, the technique of intraoral “sandwich” osteotomy works well in feminizing males. Interpositional implants of hydroxylapatite or autogenous bone may be used.^1^,^3^,^6^ Often, reduction of the buccal fat pad helps accentuate the malar process.

*Reduction rhinoplasty*. Rhinoplasty is often needed to feminize a large masculine nose with a dorsal hump. The male dorsum is relatively straight and often humped with very little supratip break. In young women, the tip has more projection and is raised and greater concavity is also seen on the dorsum; accomplishing this via rhinoplasty often produces dramatic results with a very feminizing effect.^1^,^3^–^5^

*Forehead feminization*. This may be done via rounding off the supraorbital prominences or augmentation cranioplasty. To feminize the supraorbital area, the eyebrows should be raised so that they are just above the supraorbital rim, with the high point approximately at the midpoint between the lateral limbus and the outer canthus. The feminized brow is also arched, in contrast to the flatness often seen in the male brow. In addition, the male brow lies lower on the orbital rim and forms a “T” with the nose and also exhibits greater forehead bossing, which could be due to either bony prominence or upper lid fat pads. Both of these are reduced surgically. The hairline may also be lowered to give a more feminine appearance.^1^,^3^

*Chondroplasty*. A prominent Adam's apple interferes with the acceptance as a female; thyroid cartilage reduction or chondroplasty is done in such cases.^1^,^3^,^11^

*Laser hair removal*. Epilation is essential to make the male face look feminine.^1^,^3^

## RELIGIOUS AND POLITICAL ASPECTS

Sex reassignment surgery (SRS) was uncommon in our country before 1990 because this topic was never put to debate and there was no decree on the subject. However, in 1991 this issue was put to debate by the religious clergy and was decreed to be a permitted therapeutic procedure by the highest religious authority; a *fatwa* (religious decree) was issued in this regard. Since then, the incidence of this procedure has been on the increase and does not relate to politics or the political atmosphere whatsoever; rather, it is a therapeutic issue. Religious decrees concerning therapeutic policies are issued via high-ranking religious authorities (*Mujtahids*) as are social issues. As science advances, decrees may be issued or modified to conform and adapt religious doctrines to the present era and to manage emerging problems in contemporary society. Today, SRS, when deemed necessary by the patient's doctor, has become accepted in our country as well as many other countries in properly planned cases to suit the new gender. Doctors are authorized to carry out such procedures when deemed necessary by the treatment team and when this is envisioned to be in the best interests of the patient.

## CONCLUSION

A combination of surgical procedures is required for male feminization to alter the masculine hard and soft tissue features. Application of these surgical techniques can produce favorable results. The available data have shown that in properly selected patients, surgery yields significant improvements in lifestyle, social relationships, self-esteem, body image, employment status, and sexual adjustment. Most investigators describe the outcome of SRS as satisfactory.^1^,^3^,^4^,^8^ However, SRS is no panacea; alleviation of gender problems does not automatically lead to a happy life.^4^ A general practitioner, psychiatrist, internist, endocrinologist, gynecologist, urologist, plastic surgeon, and facial surgeon are involved simultaneously in such cases. Proper patient selection is important in treating transsexuals because gender reassignment constitutes rehabilitation rather than a cure. In this respect, psychosocial guidance and therapy is of paramount importance.^4^,^8^ Although our patients were satisfied with their improvement in appearance with these standard set of operations, conclusions as to these effects cannot be drawn. Even if feminization is considerable, the degree of improvement these procedures have on lifestyle remains to be assessed.

## Figures and Tables

**Table 1:** Comparison of facial features and parameters in males and females

No.	Facial feature	Comparison	Difference
1	Nasofrontal angle	Greater in females	134 vs 131 degrees
2	Chin	More pointed in females	Males have broader chins
3	Supraorbital bossing	Less in females	
4	Zygomatic prominence	More pronounced in females	
5	Zygomatic width	Less in females	130 mm vs 137 mm
6	Bigonial width	Less in females	91 mm vs 97 mm
7	Mouth width	Less in females	50 mm vs 53 mm
8	Intercanthal distance	Less in females	31 mm vs 33 mm
9	Facial height	Less in females	112 mm vs 121 mm
10	Lower facial height	Less in females	66 mm vs 72 mm
11	Eyebrow position	Raised just above the supraorbital rim in females and arched	Slightly lower in males and flat and forms a “T” with the nose
12	Hairline position	Lower in females	
13	Nasal dorsum	Slightly concaved in females	Relatively straight in males
14	Nasolabial angle	Greater in females	107° vs 90°
15	Columella show	Greater in females	3 mm vs 1 mm
16	Alar base width	Less in females	2–3 mm less
17	Facial skin	Less hair-bearing in females; softer with smaller pore size	
18	Incisor tooth-show lips in repose	Greater in females	3 mm vs 1 mm
19	Larynx	Less prominent in females	
20	Nasal tip	More projected in females	
